# First-Generation Synthetic Cathinones Produce Arrhythmia in Zebrafish Eleutheroembryos: A New Approach Methodology for New Psychoactive Substances Cardiotoxicity Evaluation

**DOI:** 10.3390/ijms241813869

**Published:** 2023-09-08

**Authors:** Elisabet Teixidó, Clara Riera-Colomer, Demetrio Raldúa, David Pubill, Elena Escubedo, Marta Barenys, Raul López-Arnau

**Affiliations:** 1 GRET and Toxicology Unit, Department of Pharmacology, Toxicology and Therapeutic Chemistry, Faculty of Pharmacy and Food Sciences, University of Barcelona, 08028 Barcelona, Spain; 2 Institute of Nutrition and Food Safety, University of Barcelona (INSA-UB), 08921 Santa Coloma de Gramenet, Spain; 3 Department of Pharmacology, Toxicology and Therapeutic Chemistry, Faculty of Pharmacy and Food Sciences, Pharmacology Section, Institute of Biomedicine (IBUB), University of Barcelona, 08028 Barcelona, Spain; 4 Institute for Environmental Assessment and Water Research (IDAEA-CSIC), 08034 Barcelona, Spain; 5 German Centre for the Protection of Laboratory Animals (Bf3R), German Federal Institute for Risk Assessment (BfR), 10589 Berlin, Germany

**Keywords:** new psychoactive substances, synthetic cathinones, zebrafish, cardiotoxicity, QT-interval

## Abstract

The increasing number of new psychoactive substances (NPS) entering the illicit drug market, especially synthetic cathinones, as well as the risk of cardiovascular complications, is intensifying the need to quickly assess their cardiotoxic potential. The present study aims to evaluate the cardiovascular toxicity and lethality induced by first-generation synthetic cathinones (mephedrone, methylone, and MDPV) and more classical psychostimulants (cocaine and MDMA) in zebrafish embryos using a new approach methodology (NAM). Zebrafish embryos at 4 dpf were exposed to the test drugs for 24 h to identify drug lethality. Drug-induced effects on ventricular and atrial heart rate after 2 h exposure were evaluated, and video recordings were properly analyzed. All illicit drugs displayed similar 24 h LC_50_ values. Our results indicate that all drugs are able to induce bradycardia, arrhythmia, and atrial-ventricular block (AV block), signs of QT interval prolongation. However, only MDPV induced a different rhythmicity change depending on the chamber and was the most potent bradycardia and AV block-inducing drug compared to the other tested compounds. In summary, our results strongly suggest that the NAM presented in this study can be used for screening NPS for their cardiotoxic effect and especially for their ability to prolong the QT intervals.

## 1. Introduction

**Figure 1:**
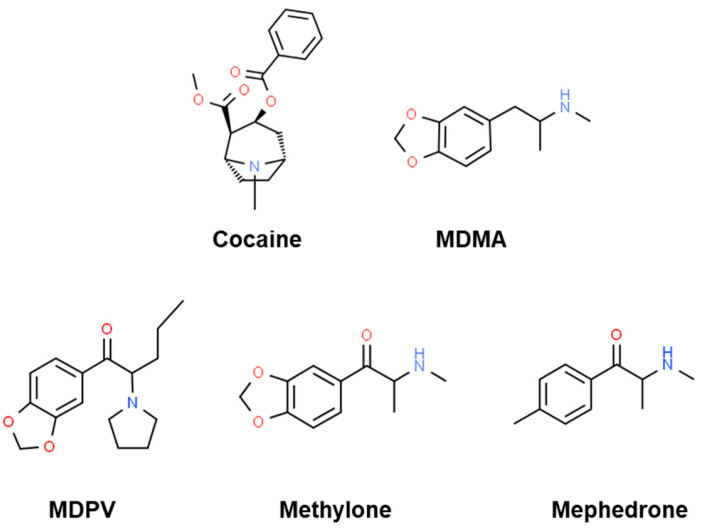
Chemical structures of cocaine and MDMA, as well as the synthetic cathinones MDPV, methylone, and mephedrone.

In the last decade, a huge number of new psychoactive substances (NPS) has broken into the illicit drug market. One of the most prevalent groups of NPS is synthetic cathinone, β-keto analogs of amphetamine derivatives, whose psychostimulant and empathogenic effects mimic classical stimulants such as cocaine or 3,4-methylenedioxy-methamphetamine (MDMA). For instance, some of the most popular synthetic cathinones are mephedrone, methylone, and 3,4-methylenedioxy-pyrovalerone (MDPV), also known as first-generation synthetic cathinones (Figure 1). On the one hand, it is known that mephedrone and methylone act as non-selective monoamine-releasing agents, increasing dopamine (DA), serotonin (5-HT) and norepinephrine (NE) concentrations in the synaptic cleft through reverse transport [1,2,3,4,5,6], similarly to amphetamine derivatives. On the other hand, MDPV acts as a potent DA transporter (DAT) and NE transporter (NET) blocker, and its mechanism of action is, therefore, more similar to the one of cocaine [2,7,8,9] (for a review, see [10]).

The effects induced by synthetic cathinones mainly include neurological, psychopathological, and cardiovascular symptoms (for a review, see [11]). In fact, several case reports of intoxication and even fatalities related to cardiovascular complications have been published [12,13,14,15,16,17]. The cardiovascular effects of synthetic cathinones in humans are similar to those induced by amphetamine derivatives and cocaine, including tachycardia; hypertension; S–T segment change, with a subsequent risk of myocardial infarction; stroke; and upon prolonged abuse, dilated cardiomyopathy [18,19,20]. A major challenge in detecting these potentially severe cardiovascular effects is the difficulty of setting up adequate animal models to study their effects within the necessary short timeframe. The need for a testing strategy allowing a quick testing response arises from the challenging reality of a continuous introduction of NPS in the market and the subsequent relatively quick disappearance/substitution of these products.

Currently, the potential cardiotoxic effects of a new drug are evaluated using whole animal in vivo studies and in vitro assays using simplified cell lines that overexpress ion channels and receptors (such as the hERG potassium channel) or animal-derived primary cardiac tissues [21]. Cell-based in vitro assays offer a quick and cost-effective approach. However, their predictive capacity is limited due to their inherent biological simplicity. Rodent animal models play a crucial role in toxicology research; however, evaluating chemical impacts in mammals entails ethical concerns and is a time-consuming and comparatively expensive process.

A new approach methodology (NAM) for cardiotoxicity evaluation alternative to testing in rodents is the use of zebrafish embryos (*Danio rerio*). Indeed, when compared with rodents, zebrafish offers advantages such as higher fecundity, faster development, and lower quantities of test chemicals required [22]. Additionally, the use of zebrafish embryos up to 5 days post-fertilization (dpf) is considered an alternative method to animal experimentation since several animal welfare regulations do not cover embryonic life stages until independent feeding [23,24]. One remarkable feature of zebrafish is the transparency of its larval stages, allowing for easy visualization and evaluation of developmental processes. This transparency is particularly advantageous when examining the cardiovascular system, as it facilitates the rapid assessment of heart rate and rhythm. Within just 26 h of fertilization, zebrafish already have a beating heart equipped with a diverse range of ion channels and a functional metabolism [25]. Zebrafish have a similar electrocardiogram to humans with clearly distinguishable P, QRS and T waves [26]. Zebrafish also possess a hERG orthologue, which is exclusively expressed in the heart and exhibits functional similarity to its mammalian counterpart [27]. Notably, hERG blockade in zebrafish can be detected without performing an electrocardiogram since it leads to bradycardia or decoupling of atrioventricular (AV) beat rates, resulting in a 2:1 AV block [28]. Although the cardiovascular system is among the earliest systems to develop, the zebrafish embryo relies on passive diffusion of oxygen from water during the initial stages. Therefore, larval zebrafish demonstrate a remarkable tolerance to significant disruptions in cardiac function, enabling them to survive for a period of four to five days without functional circulation [29]. In addition, zebrafish embryos offer important features for drug screening, such as being housed in multi-well plates, requiring small volumes and amounts of compounds for testing but also offering the potential for medium to high-throughput screening of different endpoints [30,31]. In fact, as a vertebrate, zebrafish have conserved pharmacological targets and nervous system structures comparable with mammals, including humans [32,33]. Thus, the zebrafish model has emerged as a valuable system for understanding the effects of certain toxins and drugs on cardiovascular regulation [28,34,35].

The steadily increasing number of NPS entering the illicit drug market [36], as well as the likely use of some cathinone derivatives as therapeutical agents when the time comes, is intensifying the need to assess their safety and efficacy, especially regarding their cardiotoxic effects. Moreover, since the majority of synthetic cathinone overdose cases involve polydrug use, including combinations of this class of NPS [15,16], it is difficult to identify the risk of particular synthetic cathinones based on clinical data alone. In this sense, preclinical evaluations help to identify and clarify the risks of individual synthetic cathinones. Thus, the present study aims to evaluate the cardiovascular toxicity and lethality induced by synthetic cathinones in 4-day-old zebrafish eleutheroembryos after 2 h and 24 h of exposure. This developmental stage is chosen because it represents a developmentally relevant stage when the zebrafish begin to exhibit a chronotropic response to adrenergic agonists [37] and have a complete dopaminergic system [38], allowing for meaningful assessment. Moreover, such effects are compared with those of classical psychostimulants such as cocaine and MDMA.

## 2. Results

### 2.1. Twenty-Four-Hour Acute Toxicity Effects on Zebrafish Embryos

As a first step, zebrafish embryos at 4 dpf were exposed to the test drugs for 24 h to identify lethal concentration ranges (Figure S1) and calculate 24 h LC_50_ and Hill slope based on the drug lethality curves (Table 1). All illicit drugs were lethal in zebrafish embryos within 24 h at the tested concentrations and displayed similar 24 h LC_50_ values ranging from 126.6 to 179.8 µM. Hill slope factor was approximately 3 and 7.5 times higher for MDPV and methylone, respectively, than for the other illicit drugs, indicating a much higher lethality increase derived from a fixed concentration increase for these two substances, especially for methylone. The abnormalities detected in the embryos following exposure to drugs (MDPV and MDMA) were mainly related to disruption in their movement (Table S1).

### 2.2. Drug-Induced Effects on Heart Rate after 2 h Exposure

**Figure 2:**
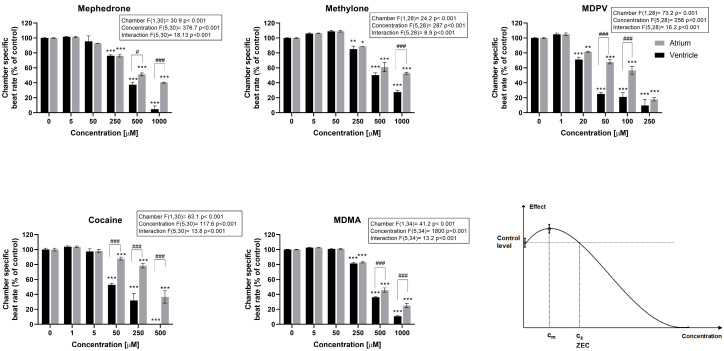
Chamber-specific beat rate of zebrafish embryos exposed to the five illicit drugs after 2 h. All values represent the mean normalized beat rate versus control ± standard error of the mean (SEM) (*n* = 10–12 embryos per clutch, at least three clutches were analyzed; * *p* < 0.05, ** *p* < 0.01, *** *p* < 0.001 compared to control values, # *p* < 0.05, ### *p* < 0.001 compared between heart chambers (atrium and ventricle)). Two-way ANOVA statistical results are displayed in the graphs.

Once lethal concentration ranges were identified, drug-induced effects on ventricular and atrial heart rate after 2 h exposure were evaluated. The concentrations tested were not lethal after 2 h exposure but included concentrations inducing lethality after 24 h to ensure that the short-term effects of these lethal concentrations could be captured. All drugs induced changes in the heart rate in a concentration-dependent manner (Figure 2 and Supplementary Figure S2), with significant effects in at least three of the concentrations tested. Interestingly, with the given EC_50_ and CI_95_ (Table 2), it was possible to distinguish a rank of potency groups among the tested substances, which was similar but not exactly equal for ventricular bradycardia:

MDPV ≈ cocaine >> mephedrone ≈ MDMA ≈ methylone

than for atrial bradycardia:

MDPV >> MDMA ≈ cocaine ≈ mephedrone >> methylone.

In both cases, MDPV was among the group of most potent bradycardia-inducing drugs, while methylone was in the group of the least potent ones. In fact, MDPV and cocaine, which were ranked among the group of the most potent or second most potent compounds, induced ventricular bradycardia from 20 µM and 50 µM, respectively. In contrast, methylone, mephedrone and MDMA only produced a significant decrease in ventricular heart rate from a concentration of 250 µM (Figure 2), thus showing that an LOAEC-based ranking would lead to the same potency pattern as the EC_50_ (CI_95_)-based ranking for ventricular bradycardia (MDPV ≈ cocaine >> mephedrone = MDMA = methylone).

**Figure 3:**
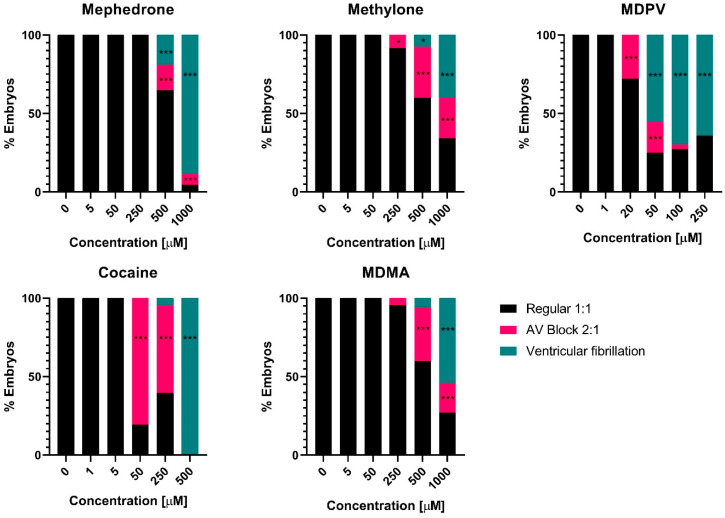
Graphical representation of the percentage of embryos exposed to each drug concentration presenting AV block (ventricle beating half as often as the atrium), ventricular fibrillation (less than 2.5 times the atrial rate), or none of these effects (regular beating heart 1:1). Fisher’s Exact test * *p* < 0.05, *** *p* < 0.001.

Additionally, EC_50_ values of MDPV and cocaine were 3.4- and 5.7-fold more potent in inducing bradycardia in the ventricle than in the atria (Table 2), revealing their higher potency to induce atrioventricular (AV) block compared to the other tested drugs. For each drug, there was a specific window concentration that produced, at some point, an AV block in the embryos (Figure 3). Atrioventricular conduction alterations progressively aggravated, leading to a significant lack of coordination in the contraction of atrial and ventricular cardiomyocytes. However, MDPV and cocaine provoked an AV block from concentrations 20 and 50 µM, respectively. In fact, at 50 µM, MDPV was inducing ventricular fibrillation (AV ratio > 2.5) in more than half of the embryos tested (Figure 3). Ventricular fibrillation was also found in all embryos at 500 µM of cocaine, while for mephedrone and MDMA, there was no AV block detected until 500 µM, and methylone exhibited AV block from 250 µM on (Figure 3).

**Figure 4:**
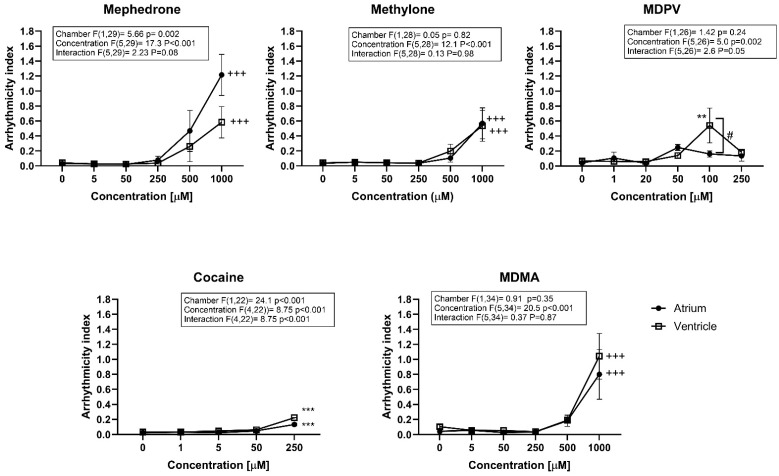
Arrhythmicity index (AI) of zebrafish embryos treated with the illicit drugs for 2 h. AI was calculated as the heart period standard deviation normalized to the median heart period. Data points represent the average AI ± standard error of the mean (SEM) (*n* = 10–12 embryos per clutch, at least three clutches were analyzed; ** *p* < 0.01, *** *p* < 0.001 compared to control AI values, # *p* < 0.05 compared between heart chambers (atrium and ventricle)). Two-way ANOVA statistical results are displayed in the graphs. Data from embryos exposed to 500 µM of cocaine had no ventricle heartbeat; therefore, no AI could be calculated. For that reason, two-way ANOVA was performed considering up to 250 µM of cocaine. For drugs with no statistically significant interaction, significant comparisons between control AI values and treated are displayed, +++ *p* < 0.001.

A third parameter calculated from the chamber-specific beat rates measured was the heart rate variability (HRV), which was measured using the AI. In this case, the effects of concentration and chamber specificity were evaluated by means of a two-way ANOVA. For MDPV and cocaine, an interaction between concentration and chamber effect was observed (interaction *p* < 0.001), which is not surprising after the observation that they induced effects with different potency in the atrium and the ventricle, mentioned in the previous paragraph. For the other three compounds (which were not detected to have different potency in the atrium and ventricle in the beat rate), no interaction between concentration and chamber was observed. In fact, for MDPV, there was a statistically significant difference between the AI of the atrium and ventricle at 100 µM. However, in all cases, zebrafish embryos exposed to the test compounds for 2 h displayed a significant increase in AI depending on concentration for both ventricle and atrium, but only at the highest tested concentrations when ventricular fibrillation was already established (Figure 4). These results indicate that all drugs are able to induce arrhythmia, but only MDPV induces a different rhythmicity change depending on the chamber. A ranking based on AI LOAECs would lead again to the same potency groupings (MDPV ≈ cocaine >> mephedrone = MDMA = methylone); however, all LOAECS would be 4 to 5 times higher than the ones described for bradycardic effects.

Although, in general, the main effect observed for all compounds was this bradycardia (or inhibitory effect on the beat rate), the first concentration tested for all illicit drugs resulted in a higher heart rate than the one of the untreated control embryos, an effect that was not significant in any case when analyzed with the two-way ANOVA considering the whole concentration-range tested. Despite not being significant, this repeated observation was considered relevant because, in clinical approaches, tachycardia is described at short times after recreational exposures, while bradycardic effects appear later in time or at higher (lethal) doses [39]. In view of this tendency, a proof-of-concept evaluation was performed to investigate if the model would also be suitable for detecting tachycardic effects of NPS after even shorter exposure times (10 min) and lower concentrations (all drugs tested at 10 µM; Figure S3).

**Figure 5:**
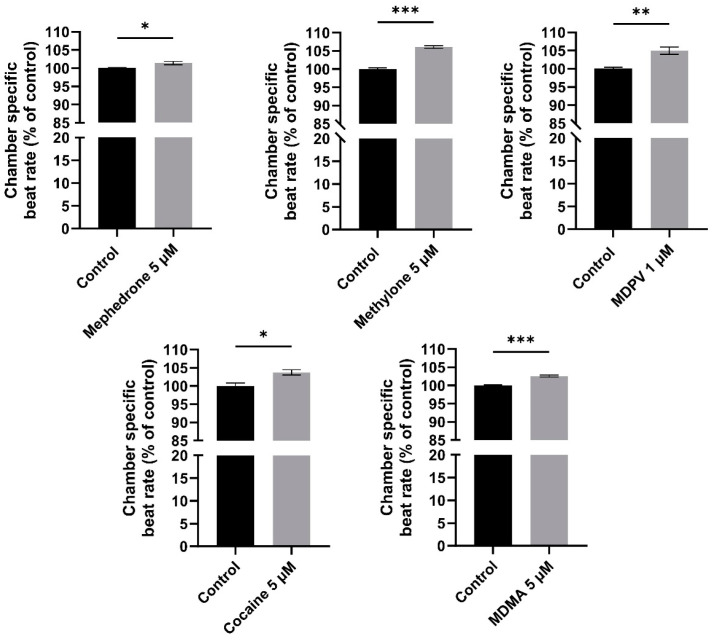
Ventricular beat rate of zebrafish embryos exposed to the Cm of the five illicit drugs after 2 h. All values represent the mean normalized beat rate versus control ± standard error of the mean (SEM) (*n* = 10–12 embryos per clutch, at least three clutches were analyzed; * *p* < 0.05, ** *p* < 0.01, *** *p* < 0.001). *t*-test statistical results are displayed in the graphs.

Indeed, when even a shorter exposure time was studied, significantly increased beat rates were detected for cocaine but not for the other drugs. Since identifying the exact combination of exposure-time and exposure-concentration of each illicit drug producing tachycardia would require a very high number of exposure scenarios, a different approach was proposed, taking into account that the response observed after the 2 h exposure to the drugs could be, in fact, a non-monotonic J-inverted shaped curve. Following the approach described in [40] and detailed in Section 4.6, we performed a *t*-test between the control group and the Cm concentration of each illicit drug (Figure 5). Considering that almost all Cms were occurring at in vivo relevant concentrations after recreational consumption of the drugs, in case two concentrations were identified between control and ZEC, the one with in vivo relevance was selected for comparison. Importantly, in all evaluations performed, the ventricular rate was significantly increased at Cm compared to the control, thus showing that the zebrafish embryo method is also able to detect the tachycardic effects produced by these drugs (Figure 5).

## 3. Discussion

Here, we present the successful application of the zebrafish embryo model as a NAM for cardiotoxicity screening of NPS. We evaluated the systemic toxicity (lethality) and cardiotoxicity of a selected group of first-generation synthetic cathinones as well as classical psychostimulants (i.e., cocaine and MDMA), including parameters like ventricle and atrium beat rate, AV block, ventricular fibrillation, and AI. This method has already been successfully applied in the past to evaluate the cardiotoxicity of different families of drugs and environmental pollutants [41,42], but to the best of our knowledge, this is the first time to be shown that it can also be used to evaluate the cardiotoxicity of NPS.

This application is highly relevant, given the constant appearance of NPS with unknown cardiotoxicity potential in the market and the concerns these drugs entail at the clinical level [36]. Testing for their cardiotoxicity or their lethal potential by means of classical in vivo methods is too slow for the speed of the real market (besides being too expensive and ethically debatable). In fact, in vivo examination of synthetic cathinones lethality is very limited in the literature. Piao and co-workers (2015) found that methylone has an LD_50_ value slightly lower than methamphetamine or MDMA in mice [43]. This was also the case in our zebrafish LC_50_ values, but, in general, NPS induced lethality at a similar range than the classical psychostimulants cocaine and MDMA, thus pointing to a similar health threat by this class of compounds. The only particularly higher threat observed in some NPS, MDPV and methylone was a comparatively much higher Hill slope in their concentration–response curves, pointing to a higher potential of accidentally reaching lethal doses by smaller dose increases. However, since zebrafish embryos are able to survive for at least one week without a functional heart, as embryos rely on passive diffusion of oxygen from water to meet their respiratory needs [29], lethality induced by these chemicals in the exposed embryos may not be directly connected to their cardiotoxicity.

**Figure 6:**
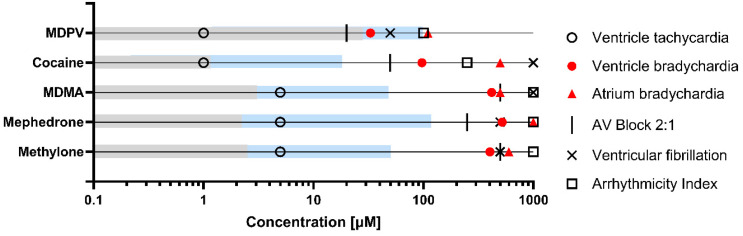
Schematic overview of the cardiotoxic effects evaluated in the zebrafish embryo model for MDPV, cocaine, MDMA, mephedrone and methylone. The following values obtained from the zebrafish experiments have been represented for each endpoint: ventricle tachycardia (LOAEC), ventricle bradychardia (EC_50_), atrium bradychardia (EC_50_), AV block 2:1 (LOAEC), ventricular fibrillation (LOAEC), arrythmicity index (LOAEC). Grey areas represent in vivo relevant concentrations in humans in blood/plasma after recreative doses described in the literature [47,48], while blue areas represent in vivo relevant concentrations in humans in blood/plasma after lethal doses described in the literature [48,49,50,51].

The concentration range evaluated in our study includes concentrations achieved after recreational doses in humans, as well as relevant concentrations after overdoses (lethal doses) and even higher non-realistic concentrations (Figure 6). This wide range allows for a better characterization of the effects than only testing the lower human exposure relevant concentration range by achieving EC_50_s for a later comparison of the potencies. The cardiotoxicity analysis of the psychostimulants tested has identified some common effects of all these drugs, such as the induction of bradycardia, AV block, irregular arrhythmia, and ventricular fibrillation and arrest at the highest concentrations. All these effects are consistent with the response of zebrafish embryos to QT-prolonging drugs [27]. Many psychoactive agents have been classified as QT-prolonging drugs, increasing the length of the repolarization phase of the cardiac action potential, an effect finally resulting in a life-threatening arrhythmia known as torsade de pointes (TdP). Cocaine, for instance, prolongs QT intervals by blocking voltage-gated potassium channels, an effect resulting in ventricular arrythmias, including TdP and AV block [44]. Interestingly, in this study, cocaine and MDPV were the most potent drugs inducing ventricular bradycardia and AV block, two of the markers of exposure to QT-prolonging drugs. In fact, these two substances share a similar mechanism of action, acting both as potent DAT blockers [2]. MDMA has been reported to increase blood pressure and heart rate, and some cases of cardiac arrhythmia have been reported after consumption of this empathogen drug [45]. Interestingly, all synthetic cathinones tested also showed cardiotoxic effects, including QT-prolonged intervals resulting in TdP [46]. Moreover, decreased beat rate (bradycardia-like effect) and increased field potential duration (prolonged QT interval-like effect) have been reported in human-induced pluripotent stem cell (hiPSC)-derived cardiomyocytes exposed to cocaine, MDMA, and MDPV [47].

Regarding the mechanism by which these substances affect cardiovascular function, and as stated before, it is known that cocaine can have a direct effect by blocking potassium, sodium, and calcium channels (i.e., hERG), prolonging QT interval, and subsequently inducing TdP, which can lead to sudden cardiac death [52,53]. However, amphetamine and MDMA do not share such ion channel-blocking properties with cocaine, pointing to an indirect cardiovascular effect via the sympathetic nervous system by increasing circulating NE levels [54,55]. Although the specific mechanism by which synthetic cathinones affect cardiac function has not been fully elucidated, it is widely known that they are able to inhibit monoamine reuptake, including NE [1,3,4,5,7,8], which may lead to an indirect cardiovascular effect. Moreover, synthetic cathinones may also have direct effects on cardiovascular function, as reported by [47].

In this regard, the method presented here is able to detect both effects, increase and decrease in beat rate, which appear at different concentration ranges. Importantly, the tachycardic effects occurred at concentrations with human in vivo relevance after consumption of recreational or lethal doses of all tested drugs. This is a clear advantage compared to the human hiPSC-derived cardiomyocytes test, where only bradycardic effects could be detected [47]. The authors discussed it as a consequence of the method being able to detect only the direct effects of the drugs. In the zebrafish method, since it is based on a whole organism, indirect effects also play a role in the response, which could be an explanation for why we could detect them and would support the hypothesis that tachycardic effects are mediated by indirect mechanisms.

Despite their limitations, both methods include relevant features and offer valuable possibilities; the hiPSC-based method has a higher human relevance and is more suitable for key event (KE) evaluation, while zebrafish includes whole organism relevance, offers the possibility to evaluate adverse outcomes (AO) and is faster and cheaper. Thus, a small battery of in vitro methods combining hiPSC-cardiomyocytes and zebrafish evaluation would be highly valuable for a thorough characterization of the cardiotoxic effects of NPS.

On the other hand, if what is needed is a single fast screening assay, ventricle beat rate EC_50_ determination is, among all parameters we evaluated, the one offering the most reliable information from several points of view: compared to AI LOAEL, ventricle beat rate EC_50_ was more sensitive, although the potency ranking obtained with both endpoints was the same (Figure 6). Atrium effects were also less sensitive, and the potency rank was slightly different than the one obtained by using the other parameters. Ventricle beat rate LOAEL is affected by the choice of experimental concentrations, and therefore, EC_50_ determination would be preferred. Finally, by measuring this endpoint and modulating the concentration range and/or the time of exposure, tachycardic effects could also be measured. The inclusion of atrium beat rate would be valuable to calculate AV block and ventricular fibrillation potential, which would contribute to better indicating the potential of prolonged QT interval-like effects. In case such a screening assay would be needed, it would be strongly suggested to use MDPV and methylone as reference compounds to allow grouping of the tested NPS according to their cardiotoxic potency.

A clear limitation of our approach is that measuring tachycardic effects by comparison of control and Cm effects does not offer the possibility to rank the potency of the compounds since Cms were not common for all compounds and no direct comparison can be made among them. A comprehensive combination of exposure scenarios varying concentration and time is needed for proper identification of this effect, but as a first step, we have been able to prove that the identification of this effect at in vivo relevant concentrations is possible by our method (Figure 6).

This study elucidates the significant effects on chamber-specific heartbeats and the impact of arrhythmia in embryos treated with illicit drugs. While recent advancements have demonstrated the benefits of transgenic fluorescent zebrafish lines in studying cardiac function [56], this study takes advantage of the zebrafish embryonic heart’s visibility through the translucent pericardium and is therefore easily adaptable to all facilities already having wild-type zebrafish embryos, without the need of breeding transgenic lines. By analyzing the power spectra of pixel intensities and specifically examining the “dynamic pixels”, both the heart rate and AI could be estimated. These measures prove to be essential, non-invasive markers for assessing cardiotoxicity in zebrafish.

In summary, our results strongly suggest that zebrafish eleutheroembryos can be used for screening NPS for their cardiotoxic effect and especially for their ability to prolong the QT intervals. Finally, further research is needed since the potential of synthetic cathinones for cardiotoxicity and lethality, as well as the mechanism underlying these effects, remain one of the least understood aspects of their use and abuse.

## 4. Materials and Methods

### 4.1. Chemicals and Test Solutions Preparation

Synthetic cathinones (MDPV, mephedrone and methylone) were synthesized in a racemic form as hydrochloride salts as described in [5,57]. Cocaine and MDMA were generously provided by the Spanish National Institute of Toxicology (Madrid, Spain). Drugs were dissolved in pure distilled water to prepare the stock solutions of 250 mM of each drug. Aliquots of the stock solutions were stored at −20 °C until the day of the test. Working solutions were prepared by diluting the stock solutions in Goods Buffer N-[Tris(hydroxymethyl)methyl]-3-aminopropanesulfonicacid (TAPS 5 mM, CAS 29915-38-6, Merck KGaA, Darmstadt, Germany) at pH 8.6. This buffer was selected according to the speciation profile of the drugs (pKa between 8 and 10), and after previously proving no adverse effects of this pH on survival or developmental parameters, as well as a higher effect of MDMA at pH 8.6 than at pH 7.4 in zebrafish embryos due to a higher bioavailability [58]. Because all the tested drugs are ionizable compounds, the pH influences uptake but not intrinsic toxicity [59].

### 4.2. Zebrafish Maintenance and Egg Production

Adult female and male zebrafish were obtained from a commercial supplier (ICA S.A., Barcelona, Spain) and housed separately in a closed flow-through system in standardized dilution ISO water (ISO, 1996; 2 mM CaCl_2_•2 H_2_O; 0.5 mM MgSO_4_•7 H_2_O; 0.75 mM NaHCO_3_; 0.07 mM KCl). Fish were maintained at 26 ± 1 °C under a 14:10 light:dark cycle. Maintenance of the adult colony was approved by the Ethics Committee for Animal Experimentation of the University of Barcelona (CEEA), accepted by the Department of Environment and Housing of the Generalitat de Catalunya with the license number 334/18, and according to the Generalitat de Catalunya Decree 86 214/1997 of 30 July, which regulates the use of animals for experimental and other scientific purposes.

The day before the experiments, females and males were transferred to a breeding tank (1:2). Zebrafish eggs were collected within 30 min after the onset of lights in the morning. Fertilized, non-coagulated, and synchronously divided eggs were selected using a stereomicroscope (Motic SMZ168, Motic China Group, LTD., Hong Kong, China). Eggs were transferred to a crystallization dish with ISO water at a ratio of 1:1 and incubated at 26 ± 1 °C under a 14:10 light:dark cycle until the day of the experiment.

### 4.3. Exposure to Drugs

Experiments were conducted to assess lethality after 24 h exposure and quantify drug-induced effects on heart rate after 2 h exposure. Experiments were performed on day 4 of zebrafish development (4 dpf). On the day of the experiment, eleutheroembryos (hereafter embryos) were distributed into a 6-well plate (Thermo-Fisher Scientific, Madrid, Spain) by adding 12 embryos into each well with 5 mL of test solution. Embryos were exposed by immersion at 20 min intervals so that they could be mounted individually by ensuring equal exposure times in all groups. Each drug was tested at different concentrations (ranging from 1 to 1000 µM) in parallel with a control group (Goods buffer TAPS alone). The concentration range used was based on previous experiments with MDMA [58].

### 4.4. Preparation of Embryos and Video Recording

To avoid the movement of zebrafish embryos during recording, embryos were anesthetized by adding tricaine to exposure medium (0.08 mg/mL) and mounted in 3% methylcellulose (without tricaine) on double depression slides (Paul Marienfeld GmbH & Co. KG, Lauda-Königshofen, Germany) after drug exposure time. The concentration of tricaine used was similar to that used in other zebrafish studies and has been shown to not affect heart rate in zebrafish embryos [27,37]. The slide was then transferred to an inverted light microscope (Nikon eclipse TS100, at 400×/N.A. 0.55, Nikon Instruments Inc., Amsterdam, The Netherlands) fitted with a high-speed video camera (Exilim, CASIO, Shibuya City, Japan). Sequential images of the heart (240 frames per second (fps)) were obtained with the embryo positioned on its side from the lateral position and with a duration of 10 s. Embryos were recorded one at a time, and all experiments were consistently conducted at the same time of day, controlling room temperature variation throughout video recordings.

### 4.5. Twenty-Four-Hour Acute Toxicity Test

Embryos at 4 dpf were distributed to 6-well plates, and 10 embryos were placed into each well with 5 mL of drug test solution. Plates were incubated at 26 ± 1 °C under a 14:10 light:dark cycle for 24 h. Following 24 h exposure, endpoints used for assessing lethality (coagulation or lack of heartbeat) and morphological/functional abnormalities were recorded for each treatment group. Each drug was tested at different concentrations, and at least three independent experiments were performed.

### 4.6. Heart Rate Analysis

To quantify atrial and ventricular heart rates in zebrafish, video recordings were analyzed with ImageJ v.1.53t [60]. A plot of dynamic pixels was obtained by selecting an area of the heart ventricle or atrium with a high deviation of pixel intensity by means of the z-projection function (see Figure S4 for representative selection of heart ventricle and atrium area). The frequency as frames/beat was obtained by analyzing the waveform of dynamic pixels by the Fast Fourier Transform algorithm (FFT) using the “spectrum” function in R. Then, the resulting periodogram provides frequency information in cycles per frame, so to calculate the heart rate frequency (beats/min, bpm), the frequency values were multiplied by the frame rate (240 fps) to convert cycles per frame to cycles per second (Hz) and then multiplied by 60 to convert from Hz to bpm.

To evaluate tachycardic effects in J-inverted shaped curves, the approach described by [40] for J-shaped curves was applied. In J-inverted concentration–responses, there is an increase in the adverse effect from the control level to a maximum response at a concentration designated as Cm (dm for dose in [40]). Above this concentration, the response decreases until a result at the control level is again obtained at the ZEC (zero equivalent concentration). The adverse response continues to decrease at concentrations above the ZEC. In these cases, the difficulty lies in demonstrating the presence of an inverted J-shaped concentration–response. This requires showing an increase in the adverse effect at low concentrations because such decreases are very often much smaller in relation to the main decrease observed for adverse effects at high concentrations. As previously described, the most powerful statistical test for a J-shaped concentration–response is obtained by comparison of the response of the control group with the response at the Cm, and in the case of continuous data, ordinary *t*-tests can be conducted [40], as in this case.

**Figure 7:**
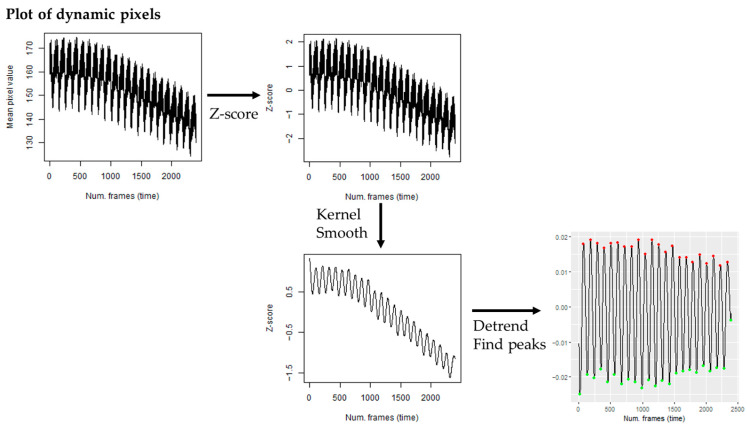
Schematic representation of the approach used to calculate the interbeat intervals (heart period) from video recordings of zebrafish embryos. Color dots in the bottom right image indicate identified peaks (local maxima, red and minima, green) in data. Methodology adapted from [61].

Heartbeat variability was determined by analysis of the interbeat intervals (heart period) (see Figure 7), similar to the analysis described in [61]. Briefly, the generated dynamic pixels plot was standardized by z-scores and smoothed with a kernel regression. Then, any linear trend was removed, the times at which local peaks occurred were determined using the ggpmisc package in R, and the differences between peak times (heart period) were calculated. The variability in the heart period can be quantified using the arrhythmicity index (AI), the heart period standard deviation normalized to the median heart period [62]. This procedure was performed automatically using a workflow in KNIME^®^ [63] with custom R scripts.

The fraction of embryos exhibiting a 2:1 AV block was determined by calculating the embryos in which the ventricle was beaten half as often as the atrium (approximately 1.8 to 2.5 times). Embryos displaying more pronounced irregular ventricular contractions (less than 2.5 times the atrial rate) were classified as having ventricular fibrillation, which, at higher concentrations, resulted in the cessation of ventricular contractions.

### 4.7. Cardiovascular Data Analysis

Concentration–response curves were derived from 2 h exposures and the 24 h acute toxicity test as effective concentrations 50 (EC_50_) and lethal concentrations 50 (LC_50_), respectively. Concentration–response curves were obtained with a sigmoidal concentration–response equation (Hill equation) using the package drc [64] in R software (v.R-3.6.1).

Statistical analyses were conducted using GraphPad Prism 9 software. For all endpoints reported as means of mean values, data were considered to have a normal distribution according to the central limit theorem (by which means of random samples from any distribution will themselves have a normal distribution) [65,66]. Statistical comparison was performed by two-way ANOVA, followed by Tukey’s test, which was conducted to assess the effect of concentration and chamber (atrium–ventricle) on heart frequency or AI. Fisher’s Exact test was used for the statistical analysis of the proportion of embryos showing AV block and ventricular fibrillation. Students *t*-test was used to analyze the ventricular beat rate of zebrafish embryos exposed to the Cm.

## Figures and Tables

**Table 1:** Twenty-four-hour acute zebrafish toxicity test (from 4 to 5 dpf) of the test compounds. LC_50_ with confidence interval 95 (CI_95_) and Hill slope obtained from the lethality concentration–response curves presented in Supplementary Materials Figure S1.

Compound	LC_50_ (CI_95_) µM	Hill Slope
Cocaine	172.9 (153.3–210.3)	2.81
MDPV	135.2 (114.6–155.7)	6.88
MDMA	179.8 (125.6–234.0)	2.65
Methylone	126.6 (92.1–161.1)	15.3
Mephedrone	147.4 (92.7–202.0)	1.86

**Table 2:** Effective concentrations (EC_50_) of atrial and ventricular heart rate after 2 h of exposure to the illicit drugs derived from the concentration–response curves presented in Supplementary Materials Figure S2.

COMPOUND	EC50 (CI95) µM ATRIUM	EC50 (CI95) µM VENTRICLE
Cocaine	>500	97.2 (29.9–129.0)
MDPV	110.5 (74.0–127.0)	32.7 (18.3–46.1)
MDMA	500 (412.8–586.1)	418.3 (359.7–476.9)
Methylone	>1000	522.3 (414.8–664.1)
Mephedrone	600.6 (522.5–693.2)	403 (377.5–423.3)

## Data Availability

The data that support the findings of this study are available from the corresponding author upon reasonable request.

## Supplementary Materials

The following supporting information can be downloaded at: https://www.mdpi.com/article/10.3390/ijms241813869/s1.
