# Supplementary material for: First-Generation Synthetic Cathinones Produce Arrhythmia in Zebrafish Eleutheroembryos: A New Approach Methodology for New Psychoactive Substances Cardiotoxicity Evaluation
Source: Int J Mol Sci. 2023 Sep 8;24(18):13869. doi: 10.3390/ijms241813869 (PMC10531093; doi:10.3390/ijms241813869)
Supplement: Supplementary file 1 [file ijms-24-13869-s001.zip › ijms-2576815-supplementary.pdf]

Supplemental data

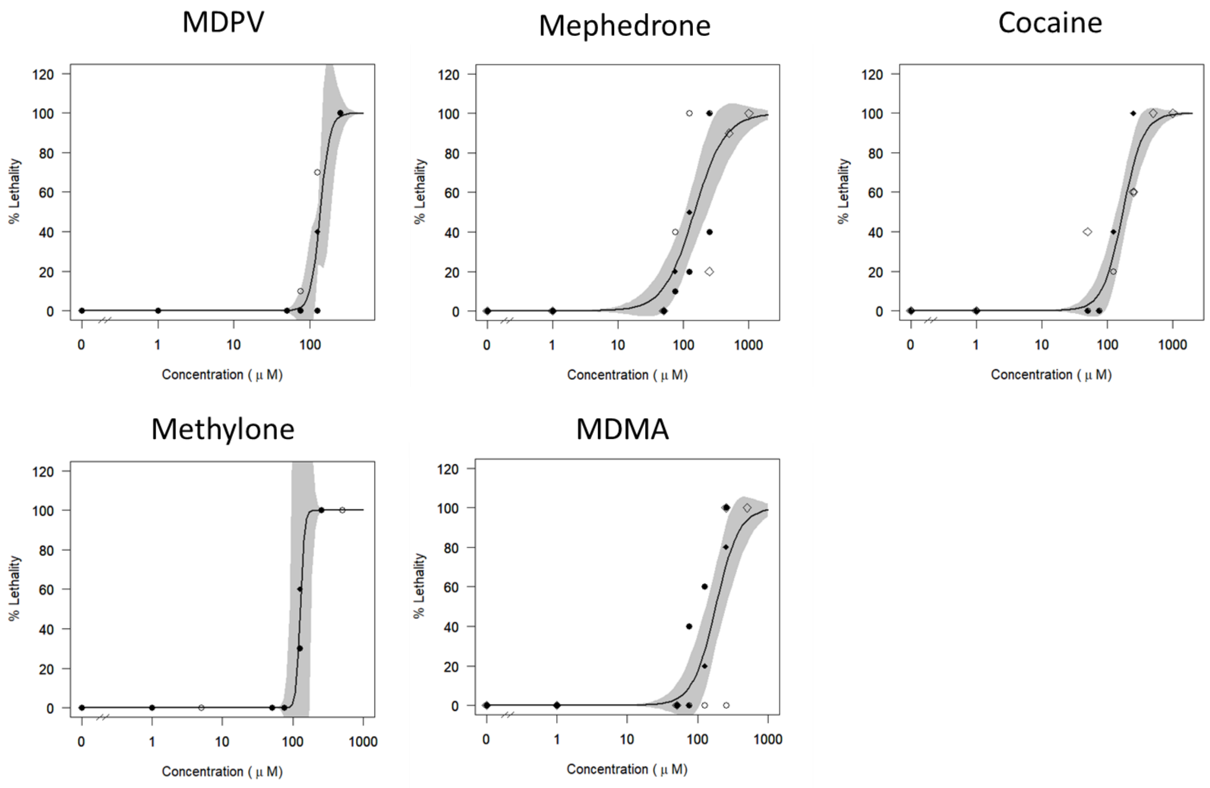

Figure S1. Drug-induced concentration-dependent lethality after 24-h exposure between 4 and 5 dpf. Each dot represents a tested concentration, different symbols represent the replicates. At least three replicates were performed.

Table S1. Drug-induced abnormalities after 24h-exposure between 4 and 5 dpf. Only concentrations with abnormal embryos are given for each drug, there were no instances of abnormalities noted in the other tested concentrations.

DRUG	ABNORMALITIES
**MDPV**	125 mM: 21% embryos with spasms.
**MEPHEDRONE**	No abnormalities observed
**COCAINE**	No abnormalities observed
**METHYLONE**	No abnormalities observed
**MDMA**	125 mM: 45.4% embryos with spasms and reduced mobility.<br>250 mM: 83.3% embryos with spasms and reduced mobility.

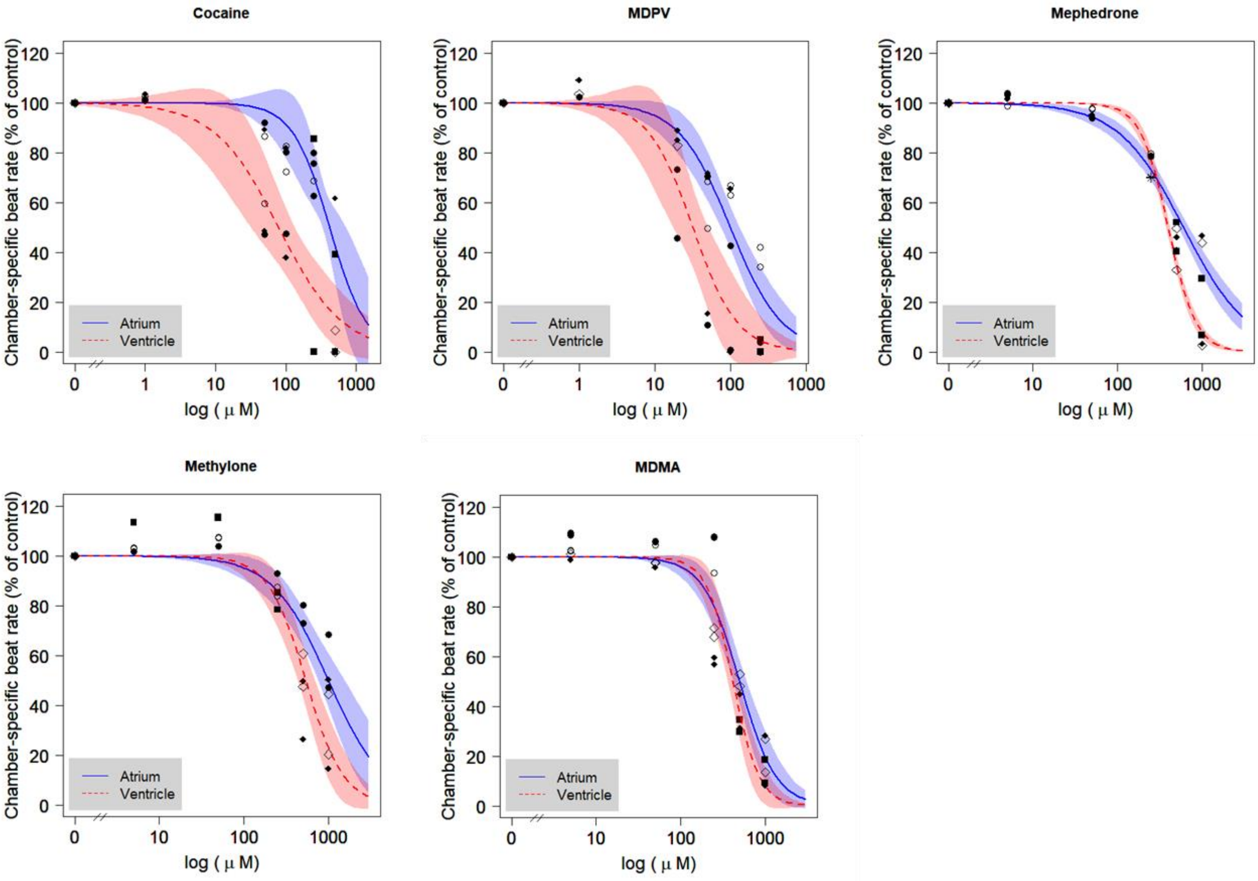

Figure S2. Concentration-response curves showing chamber-specific heart rate for the different concentrations of illicit drugs tested on 4 dpf old zebrafish larvae. Embryos were incubated for 2 hours with each drug (n=12). Data are representative of at least three independent experiments.

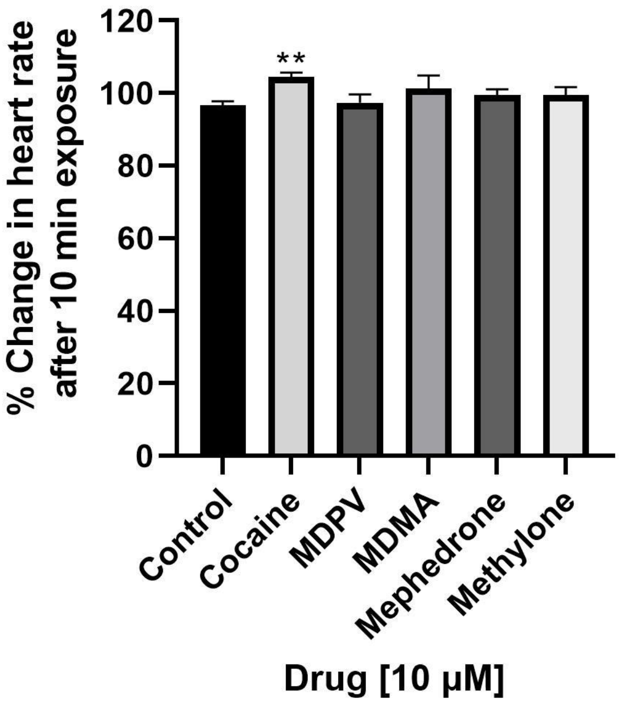

Figure S3. Effects on zebrafish heart rate after 10-minute exposure to each illicit drug at 4 dpf. Embryos at 4 dpf distributed into a 6-well plate with 5 mL of Goods buffer TAPS were anaesthetized (0.08 mg/mL tricaine) and mounted in 3% methylcellulose on double depression slide to record the basal heart rate before exposure. Embryos were exposed individually by adding 20 µL to the depression slide for 10 minutes after which heart rate was recorded again. Each drug was tested at a fixed concentration of 10 µM using 6 larvae. ** $p < 0.01$.

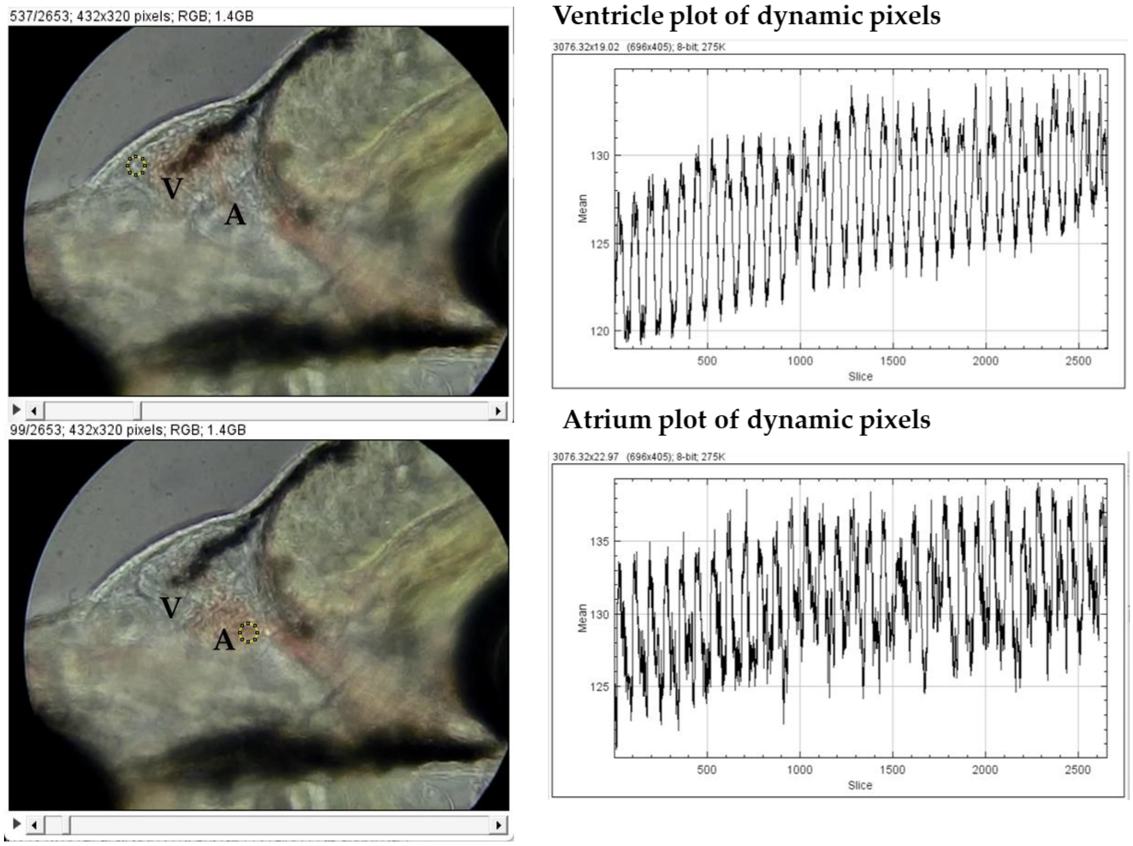

Figures S4. Representative image frames of zebrafish heart recordings depicting the ventricle (V) and atrium (A). The left image on top shows the heart during ventricle end-diastole, while the left image on bottom corresponds to the heart during atrium end-diastole. Images on the right represent the plot of dynamic pixels obtained from the z-axis profile of the selected region of the heart (yellow circle) with high deviation of pixel intensity on left stack images. The time series is used to calculate heart rate for ventricle and atrium. Analysis were performed on ImageJ v.1.53J.
